# [3]Ferrocenophan-1-one

**DOI:** 10.1107/S1600536808022927

**Published:** 2008-07-23

**Authors:** Petr Štěpnička, Ivana Císařová

**Affiliations:** a Department of Inorganic Chemistry, Faculty of Science, Charles University, Hlavova 2030, 12840 Prague, Czech Republic

## Abstract

The crystal structure of [3]ferrocenophan-1-one, [Fe(C_13_H_12_O)], has been redetermined at 150 K. The tethered cyclo­penta­dienyl (Cp) rings are tilted by 9.39 (18)° and assume an eclipsed conformation. The 1-oxopropane-1,3-diyl bridge has a pseudo-envelope conformation with the C=O group deviating by as much as 22.5 (2)° from coplanarity with its attached Cp ring.

## Related literature

For an overview of the chemistry of ferrocene, see: Štěpnička (2008 ▶). For the preparation of the title compound, see: Turbitt & Watts (1972 ▶). For its crystal structure at room temperature, see: Jones *et al*. (1965 ▶). For an introductory review on the chemistry of ferrocenophanes with carbon bridges, see: Heo & Lee (1999 ▶).
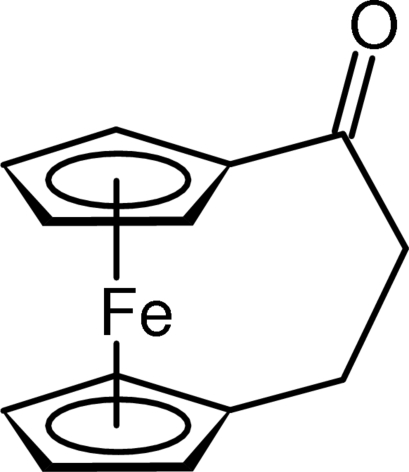


## Experimental

### 

#### Crystal data

[Fe(C_13_H_12_O)]
*M* *_r_* = 240.08
Monoclinic, 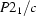

*a* = 5.77450 (10) Å
*b* = 7.3303 (2) Å
*c* = 22.8596 (6) Å
β = 93.242 (2)°
*V* = 966.07 (4) Å^3^
*Z* = 4
Mo *K*α radiation
μ = 1.52 mm^−1^
*T* = 150 (2) K
0.38 × 0.30 × 0.28 mm

#### Data collection

Nonius KappaCCD diffractometer
Absorption correction: multi-scan (*SORTAV*; Blessing, 1995 ▶) *T*_min_ = 0.579, *T*_max_ = 0.660
13397 measured reflections
2223 independent reflections
2102 reflections with *I* > 2σ(*I*)
*R*_int_ = 0.032

#### Refinement

*R*[*F* ^2^ > 2σ(*F* ^2^)] = 0.038
*wR*(*F* ^2^) = 0.096
*S* = 1.31
2223 reflections
136 parameters
H-atom parameters constrained
Δρ_max_ = 0.53 e Å^−3^
Δρ_min_ = −0.38 e Å^−3^

### 

Data collection: *COLLECT* (Nonius, 2000 ▶); cell refinement: *SCALEPACK* (Otwinowski & Minor, 1997 ▶); data reduction: *DENZO* (Otwinowski & Minor, 1997 ▶) and *SCALEPACK*; program(s) used to solve structure: *SIR97* (Altomare *et al.*, 1999 ▶); program(s) used to refine structure: *SHELXL97* (Sheldrick, 2008 ▶); molecular graphics: *PLATON* (Spek, 2003 ▶); software used to prepare material for publication: *PLATON* and *publCIF* (Westrip, 2008 ▶).

## Supplementary Material

Crystal structure: contains datablocks I, global. DOI: 10.1107/S1600536808022927/dn2366sup1.cif

Structure factors: contains datablocks I. DOI: 10.1107/S1600536808022927/dn2366Isup2.hkl

Additional supplementary materials:  crystallographic information; 3D view; checkCIF report

## Figures and Tables

**Fig. 1.**
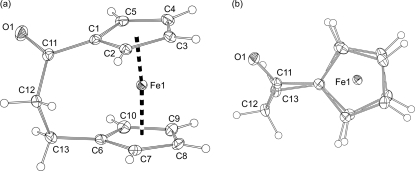
Side (a) and top (b) views of I showing the atom numbering scheme and displacement ellipsoids for the non-H atoms at the 30% probability level.

## supplementary crystallographic information

### Comment

The chemistry of ferrocene has received considerable attention because of its widespread applications ranging from material science to biomedicine. A particularly successful area is undoubtedly catalysis with ferrocene ligands (Štěpnička, 2008). A number of ferrocene ligands has been prepared a studied as ligands for transtion metal-mediated reactions. Among the numerous ligands reported to date, a specific class is constituted by the donors whose cyclopentadienyl (Cp) rings are interconnected with a linking group (*i.e.*, ferrocenophane-type compounds (Heo & Lee, 1999)). [3]ferrocenophan-1-one (I) represent a convenient entry to such donors (Štěpnička, 2008). Because the crystal structure of I has been reported already in the middle 1960's (Jones *et al.*,  1965), we have redetermined it at 150 K in order to obtain more precise structural information.

The molecular structure of I (Figure 1) is rather unexceptional as far as interatomic distances and angles concerns. Because of spatial constraints imposed by the 1-oxapropan-1,3-diyl linker, the Cp rings are tilted by 9.39 (18)° and adopt a near-to-eclipsed conformation characterized by the torsion angle C(1)—*Cg*(1)—*Cg*(2)—C(6) of -5.6 (2)°, where *Cg*(1) and *Cg*(2) are the centroids of the Cp rings C(1–5) and C(6–10), respectively. The iron—*Cg* distances in I are: Fe1—*Cg*(1) 1.6399 (14) Å and Fe1—*Cg*(2) 1.6463 (14) Å. The aliphatic bridge assumes a pseudoenvelope conformation and its C═O bond (C11—O 1.2012 (4) Å) is displaced above its bonding Cp ring, the angle subtended by the C?O vector and the Cp ring being 22.5 (2)°.

### Experimental

The title compound was synthesized by acylation of ferrocene with acryloyl chloride in the presence of AlCl_3_ (Turbitt & Watts, 1972) and characterized by ^1^H and ^13^C{^1^H} NMR spectra. Orange-red crystals suitable for X-ray diffraction analysis were obtained by liquid-phase diffusion of hexane into a solution of the compound in dichloromethane.

### Refinement

All H-atoms were included in calculated positions and refined with d(C—H) = 0.93 Å (aromatic) and 0.97 Å (methylene) and *U*_iso_(H) = 1.2*U*_eq_(C).

### Crystal data

**Table:**

[Fe(C_13_H_12_O)]	*F*_000_ = 496
*M**_r_* = 240.08	*D*_x_ = 1.651 Mg m^−^^3^
Monoclinic, *P*2_1_/*c*	Mo *K*α radiation λ = 0.71073 Å
Hall symbol: -P 2ybc	Cell parameters from 2391 reflections
*a* = 5.77450 (10) Å	θ = 0.4–27.5º
*b* = 7.3303 (2) Å	µ = 1.52 mm^−^^1^
*c* = 22.8596 (6) Å	*T* = 150 (2) K
β = 93.242 (2)º	Block, orange–red
*V* = 966.07 (4) Å^3^	0.38 × 0.30 × 0.28 mm
*Z* = 4	

### Data collection

**Table:**

Nonius KappaCCD diffractometer	2223 independent reflections
Radiation source: fine-focus sealed tube	2102 reflections with *I* > 2σ(*I*)
Monochromator: horizontal graphite crystal	*R*_int_ = 0.032
Detector resolution: 9.091 pixels mm^-1^	θ_max_ = 27.5º
*T* = 150(2) K	θ_min_ = 1.8º
ω and π scans to fill the Ewald sphere	*h* = −7→7
Absorption correction: multi-scan(SORTAV; Blessing, 1995)	*k* = −9→9
*T*_min_ = 0.579, *T*_max_ = 0.660	*l* = −29→29
13397 measured reflections	

### Refinement

**Table:**

Refinement on *F*^2^	Secondary atom site location: difference Fourier map
Least-squares matrix: full	Hydrogen site location: inferred from neighbouring sites
*R*[*F*^2^ > 2σ(*F*^2^)] = 0.038	H-atom parameters constrained
*wR*(*F*^2^) = 0.096	*w* = 1/[σ^2^(*F*_o_^2^) + 2.5298*P*] where *P* = (*F*_o_^2^ + 2*F*_c_^2^)/3
*S* = 1.31	(Δ/σ)_max_ < 0.001
2223 reflections	Δρ_max_ = 0.53 e Å^−^^3^
136 parameters	Δρ_min_ = −0.38 e Å^−^^3^
Primary atom site location: structure-invariant direct methods	Extinction correction: none

### Special details

**Table:**

Geometry. All e.s.d.'s (except the e.s.d. in the dihedral angle between two l.s. planes) are estimated using the full covariance matrix. The cell e.s.d.'s are taken into account individually in the estimation of e.s.d.'s in distances, angles and torsion angles; correlations between e.s.d.'s in cell parameters are only used when they are defined by crystal symmetry. An approximate (isotropic) treatment of cell e.s.d.'s is used for estimating e.s.d.'s involving l.s. planes.
Refinement. Refinement of *F*^2^ against all reflections. The weighted *R*-factor *wR* and goodness of fit *S* are based on *F*^2^, conventional *R*-factors *R* are based on *F*, with *F* set to zero for negative *F*^2^. The threshold expression of *F*^2^ > 2σ(*F*^2^) is used only for calculating *R*-factors(gt) *etc*. and is not relevant to the choice of reflections for refinement. *R*-factors based on *F*^2^ are statistically about twice as large as those based on *F*, and *R*- factors based on all data will be even larger.

### Fractional atomic coordinates and isotropic or equivalent isotropic displacement parameters (Å^2^)

**Table:**

	*x*	*y*	*z*	*U*_iso_*/*U*_eq_	
Fe1	0.18818 (7)	0.12608 (6)	0.151598 (17)	0.01753 (13)	
O1	0.6106 (4)	0.4906 (4)	0.09480 (11)	0.0324 (5)	
C1	0.3394 (5)	0.3716 (4)	0.15759 (12)	0.0187 (5)	
C2	0.1123 (5)	0.3776 (4)	0.18047 (12)	0.0206 (6)	
H2	−0.0117	0.4491	0.1664	0.025*	
C3	0.1121 (6)	0.2545 (4)	0.22849 (13)	0.0246 (6)	
H3	−0.0127	0.2324	0.2515	0.029*	
C4	0.3337 (6)	0.1708 (4)	0.23551 (13)	0.0263 (7)	
H4	0.3781	0.0837	0.2635	0.032*	
C5	0.4752 (5)	0.2428 (4)	0.19256 (13)	0.0226 (6)	
H5	0.6293	0.2122	0.1877	0.027*	
C6	0.1582 (5)	0.0986 (4)	0.06275 (12)	0.0212 (6)	
C7	−0.0636 (5)	0.0829 (4)	0.08711 (13)	0.0228 (6)	
H7	−0.1912	0.1573	0.0782	0.027*	
C8	−0.0570 (5)	−0.0656 (4)	0.12733 (14)	0.0242 (6)	
H8	−0.1786	−0.1044	0.1493	0.029*	
C9	0.1688 (6)	−0.1445 (4)	0.12808 (14)	0.0255 (6)	
H9	0.2211	−0.2434	0.1506	0.031*	
C10	0.3001 (5)	−0.0440 (4)	0.08799 (13)	0.0240 (6)	
H10	0.4530	−0.0675	0.0796	0.029*	
C11	0.4118 (5)	0.4452 (4)	0.10113 (13)	0.0209 (6)	
C12	0.2345 (6)	0.4393 (4)	0.04970 (13)	0.0238 (6)	
H12A	0.2733	0.5291	0.0206	0.029*	
H12B	0.0819	0.4680	0.0628	0.029*	
C13	0.2334 (6)	0.2466 (4)	0.02244 (13)	0.0250 (6)	
H13A	0.1303	0.2467	−0.0126	0.030*	
H13B	0.3882	0.2189	0.0107	0.030*	

### Atomic displacement parameters (Å^2^)

**Table:**

	*U*^11^	*U*^22^	*U*^33^	*U*^12^	*U*^13^	*U*^23^
Fe1	0.0204 (2)	0.0150 (2)	0.0171 (2)	0.00020 (17)	−0.00002 (14)	−0.00078 (16)
O1	0.0253 (11)	0.0364 (14)	0.0359 (13)	−0.0055 (10)	0.0037 (9)	0.0061 (11)
C1	0.0220 (13)	0.0145 (12)	0.0194 (13)	−0.0010 (11)	−0.0012 (10)	−0.0025 (11)
C2	0.0256 (14)	0.0172 (13)	0.0189 (13)	0.0012 (12)	0.0003 (11)	−0.0038 (11)
C3	0.0315 (16)	0.0269 (16)	0.0155 (13)	−0.0018 (13)	0.0037 (11)	−0.0037 (12)
C4	0.0352 (17)	0.0240 (15)	0.0189 (14)	−0.0010 (13)	−0.0057 (12)	0.0010 (12)
C5	0.0225 (14)	0.0201 (14)	0.0242 (14)	−0.0009 (12)	−0.0066 (11)	−0.0020 (12)
C6	0.0270 (15)	0.0193 (14)	0.0172 (13)	−0.0014 (12)	−0.0002 (11)	−0.0049 (11)
C7	0.0229 (14)	0.0241 (15)	0.0210 (14)	−0.0026 (12)	−0.0032 (11)	−0.0039 (11)
C8	0.0260 (15)	0.0204 (14)	0.0263 (15)	−0.0068 (12)	0.0016 (12)	−0.0038 (12)
C9	0.0358 (17)	0.0123 (13)	0.0284 (15)	−0.0008 (12)	0.0008 (13)	−0.0013 (12)
C10	0.0268 (15)	0.0198 (14)	0.0258 (15)	0.0018 (12)	0.0051 (12)	−0.0057 (12)
C11	0.0255 (15)	0.0132 (13)	0.0241 (14)	−0.0009 (11)	0.0016 (11)	0.0003 (11)
C12	0.0294 (16)	0.0217 (14)	0.0199 (14)	−0.0035 (12)	−0.0011 (12)	0.0042 (12)
C13	0.0335 (16)	0.0252 (15)	0.0164 (13)	−0.0027 (13)	0.0036 (12)	−0.0028 (12)

### Geometric parameters (Å, °)

**Table:**

Fe1—C1	2.002 (3)	C4—H4	0.9300
Fe1—C2	2.015 (3)	C5—H5	0.9300
Fe1—C7	2.036 (3)	C6—C10	1.430 (4)
Fe1—C6	2.039 (3)	C6—C7	1.430 (4)
Fe1—C5	2.045 (3)	C6—C13	1.503 (4)
Fe1—C10	2.048 (3)	C7—C8	1.424 (4)
Fe1—C8	2.049 (3)	C7—H7	0.9300
Fe1—C9	2.056 (3)	C8—C9	1.425 (4)
Fe1—C3	2.063 (3)	C8—H8	0.9300
Fe1—C4	2.076 (3)	C9—C10	1.427 (4)
O1—C11	1.212 (4)	C9—H9	0.9300
C1—C2	1.440 (4)	C10—H10	0.9300
C1—C5	1.441 (4)	C11—C12	1.515 (4)
C1—C11	1.480 (4)	C12—C13	1.544 (4)
C2—C3	1.421 (4)	C12—H12A	0.9700
C2—H2	0.9300	C12—H12B	0.9700
C3—C4	1.420 (5)	C13—H13A	0.9700
C3—H3	0.9300	C13—H13B	0.9700
C4—C5	1.415 (4)		
			
C1—Fe1—C2	42.00 (12)	C4—C3—H3	125.6
C1—Fe1—C7	118.70 (12)	C2—C3—H3	125.6
C2—Fe1—C7	102.70 (12)	Fe1—C3—H3	127.7
C1—Fe1—C6	99.73 (12)	C5—C4—C3	108.2 (3)
C2—Fe1—C6	114.13 (12)	C5—C4—Fe1	68.73 (17)
C7—Fe1—C6	41.09 (12)	C3—C4—Fe1	69.44 (17)
C1—Fe1—C5	41.70 (11)	C5—C4—H4	125.9
C2—Fe1—C5	69.68 (12)	C3—C4—H4	125.9
C7—Fe1—C5	157.51 (13)	Fe1—C4—H4	127.5
C6—Fe1—C5	121.23 (12)	C4—C5—C1	108.2 (3)
C1—Fe1—C10	116.24 (12)	C4—C5—Fe1	71.13 (17)
C2—Fe1—C10	150.81 (12)	C1—C5—Fe1	67.56 (16)
C7—Fe1—C10	68.43 (12)	C4—C5—H5	125.9
C6—Fe1—C10	40.96 (12)	C1—C5—H5	125.9
C5—Fe1—C10	107.44 (13)	Fe1—C5—H5	126.9
C1—Fe1—C8	158.01 (12)	C10—C6—C7	106.8 (3)
C2—Fe1—C8	123.82 (13)	C10—C6—C13	126.6 (3)
C7—Fe1—C8	40.80 (12)	C7—C6—C13	126.4 (3)
C6—Fe1—C8	69.15 (12)	C10—C6—Fe1	69.86 (16)
C5—Fe1—C8	160.19 (13)	C7—C6—Fe1	69.36 (16)
C10—Fe1—C8	68.35 (13)	C13—C6—Fe1	121.9 (2)
C1—Fe1—C9	154.45 (13)	C8—C7—C6	108.7 (3)
C2—Fe1—C9	163.46 (13)	C8—C7—Fe1	70.09 (17)
C7—Fe1—C9	68.57 (13)	C6—C7—Fe1	69.55 (16)
C6—Fe1—C9	69.15 (12)	C8—C7—H7	125.6
C5—Fe1—C9	123.64 (13)	C6—C7—H7	125.6
C10—Fe1—C9	40.68 (12)	Fe1—C7—H7	126.3
C8—Fe1—C9	40.64 (13)	C7—C8—C9	108.0 (3)
C1—Fe1—C3	69.18 (12)	C7—C8—Fe1	69.11 (17)
C2—Fe1—C3	40.77 (12)	C9—C8—Fe1	69.95 (17)
C7—Fe1—C3	120.66 (13)	C7—C8—H8	126.0
C6—Fe1—C3	152.09 (12)	C9—C8—H8	126.0
C5—Fe1—C3	67.99 (13)	Fe1—C8—H8	126.5
C10—Fe1—C3	166.86 (13)	C8—C9—C10	107.6 (3)
C8—Fe1—C3	111.49 (13)	C8—C9—Fe1	69.41 (17)
C9—Fe1—C3	130.67 (13)	C10—C9—Fe1	69.35 (17)
C1—Fe1—C4	69.06 (12)	C8—C9—H9	126.2
C2—Fe1—C4	68.71 (12)	C10—C9—H9	126.2
C7—Fe1—C4	158.22 (13)	Fe1—C9—H9	126.6
C6—Fe1—C4	160.60 (13)	C9—C10—C6	108.9 (3)
C5—Fe1—C4	40.15 (12)	C9—C10—Fe1	69.97 (17)
C10—Fe1—C4	128.66 (13)	C6—C10—Fe1	69.17 (16)
C8—Fe1—C4	126.57 (13)	C9—C10—H10	125.6
C9—Fe1—C4	114.02 (13)	C6—C10—H10	125.6
C3—Fe1—C4	40.13 (13)	Fe1—C10—H10	126.9
C2—C1—C5	107.3 (3)	O1—C11—C1	121.3 (3)
C2—C1—C11	127.8 (3)	O1—C11—C12	121.2 (3)
C5—C1—C11	123.4 (3)	C1—C11—C12	117.0 (3)
C2—C1—Fe1	69.48 (17)	C11—C12—C13	109.1 (3)
C5—C1—Fe1	70.74 (17)	C11—C12—H12A	109.9
C11—C1—Fe1	114.28 (19)	C13—C12—H12A	109.9
C3—C2—C1	107.6 (3)	C11—C12—H12B	109.9
C3—C2—Fe1	71.43 (18)	C13—C12—H12B	109.9
C1—C2—Fe1	68.51 (17)	H12A—C12—H12B	108.3
C3—C2—H2	126.2	C6—C13—C12	114.1 (2)
C1—C2—H2	126.2	C6—C13—H13A	108.7
Fe1—C2—H2	125.4	C12—C13—H13A	108.7
C4—C3—C2	108.7 (3)	C6—C13—H13B	108.7
C4—C3—Fe1	70.44 (17)	C12—C13—H13B	108.7
C2—C3—Fe1	67.80 (16)	H13A—C13—H13B	107.6

## References

[bb1] Altomare, A., Burla, M. C., Camalli, M., Cascarano, G. L., Giacovazzo, C., Guagliardi, A., Moliterni, A. G. G., Polidori, G. & Spagna, R. (1999). *J. Appl. Cryst.* **32**, 115–119.

[bb2] Blessing, R. H. (1995). *Acta Cryst.* A**51**, 33–38. 10.1107/s0108767394005726 7702794

[bb3] Heo, R. W. & Lee, T. R. (1999). *J. Organomet. Chem.* **578**, 31–42.

[bb4] Jones, N. D., Marsh, R. E. & Richards, J. H. (1965). *Acta Cryst.* **19**, 330–336.

[bb5] Nonius (2000). *COLLECT* Nonius BV, Delft, The Netherlands.

[bb6] Otwinowski, Z. & Minor, W. (1997). *Methods in Enzymology*, Vol. 276, *Macromolecular Crystallography*, Part A, edited by C. W. Carter Jr & R. M. Sweet, pp. 307–326. New York: Academic Press.

[bb7] Sheldrick, G. M. (2008). *Acta Cryst.* A**64**, 112–122. 10.1107/S0108767307043930 18156677

[bb8] Spek, A. L. (2003). *J. Appl. Cryst.* **36**, 7–13.

[bb9] Štěpnička, P. (2008). Editor. *Ferrocenes: Ligands, Materials and Biomolecules* Chichester: Wiley.

[bb10] Turbitt, T. D. & Watts, W. E. (1972). *J. Organomet. Chem.* **46**, 109–117.

[bb11] Westrip, S. P. (2008). *publCIF* In preparation.

